# The Japanese Catheter Ablation Registry (J‐AB): Annual report in 2022

**DOI:** 10.1002/joa3.13141

**Published:** 2024-09-05

**Authors:** Kengo Kusano, Koichi Inoue, Koshiro Kanaoka, Koji Miyamoto, Yasuo Okumura, Yu‐ki Iwasaki, Kazuhiro Satomi, Seiji Takatsuki, Kohki Nakamura, Yoshitaka Iwanaga, Teiichi Yamane, Wataru Shimizu, J‐AB registry investigators

**Affiliations:** ^1^ Department of Cardiovascular Medicine National Cerebral and Cardiovascular Center Suita Japan; ^2^ Cardiovascular Division National Hospital Organization Osaka National Hospital Osaka Japan; ^3^ Department of Medical and Health Information Management National Cerebral and Cardiovascular Center Suita Japan; ^4^ Division of Cardiology, Department of Medicine Nippon University School of Medicine Tokyo Japan; ^5^ Department of Cardiovascular Medicine Nippon Medical School Tokyo Japan; ^6^ Department of Cardiology Tokyo Medical School Tokyo Japan; ^7^ Department of Cardiology Keio University School of Medicine Tokyo Japan; ^8^ Division of Cardiology Gunma Prefectural Cardiovascular Center Maebashi Japan; ^9^ Division of Cardiology Department of Internal Medicine The Jikei University School of Medicine Tokyo Japan

**Keywords:** catheter ablation, complication, J‐AB, nationwide registry

## Abstract

The Japanese Catheter Ablation (J‐AB) registry, started in August 2017, is a voluntary, nationwide, multicenter, prospective, observational registry, performed by the Japanese Heart Rhythm Society (JHRS) in collaboration with the National Cerebral and Cardiovascular Center. From January 2022, the data registration system was changed from Research Electronic Data Capture (REDCap) system to Fountayn system. The purpose of this registry was to collect the details of target arrhythmias, the ablation procedures, including the type of target arrhythmias, outcomes, and acute complications in the real‐world settings. During the year of 2022, we have collected a total of 90,042 procedures (mean age of 66.7 years and 65.9% male) from 614 participant hospitals. Detailed data were shown in Figures and Tables.

Catheter ablation has become an established therapy for the management of various cardiac arrhythmias and the procedure number has been dramatically increasing. However, little is known about the details of target arrhythmias, the ablation procedures, including the type of target arrhythmias, outcomes, and acute complications in the real‐world settings.

There are several preceding registries of catheter ablation, but the majority of which collected data from selected centers and/or selected arrhythmia and/or specified months to reveal the current status of ablations. Accordingly, we conducted a nationwide, multicenter. prospective, observational registry in Japan, named Japanese Catheter Ablation (J‐AB) registry, aiming to register all catheter ablation cases in Japan.^1^ This registry has been performed by the Japanese Heart Rhythm Society (JHRS) in collaboration with the National Cerebral and Cardiovascular Center using initially Research Electronic Data Capture (REDCap) system. From January 2022, the data registration system was changed from REDCap to Fountayn system, renamed J‐AB 2022, and the research protocol was approved by the central ethics review board of the JHRS (No. 2021001, approved on Dec 16, 2021), and participation is permitted with the approval of the director of each data‐providing institution. All participants were provided informed consent either by a written paper or by an opt out fashion and could withdraw their consent at any time. This study was also registered in the UMIN Clinical Trial Registry (UMIN 000028288) and ClinicalTrials.gov (NCT03729232). This J‐AB registry started in August 2017, since then the number of participating hospitals has increased to over 500 at the end of 2021. Annual data during the year of 2018–2021 has been already reported,^2^, ^3^, ^4^, ^5^ and now we report here the annual report of the results during the year of 2022. Figure 1 showed the cumulative procedures during the year of 2022. Figure 2 showed the number and rate of the target arrhythmias. AF ablation was the leading procedure (75.9% of all ablation procedures) in 2022, and the percentage of patients over 75 years of age was 30.4% in 2022. Patient characteristics, acute outcomes, and acute complications of all and AF procedures are shown in Tables 1, 2, 3, respectively.

**FIGURE 1:**
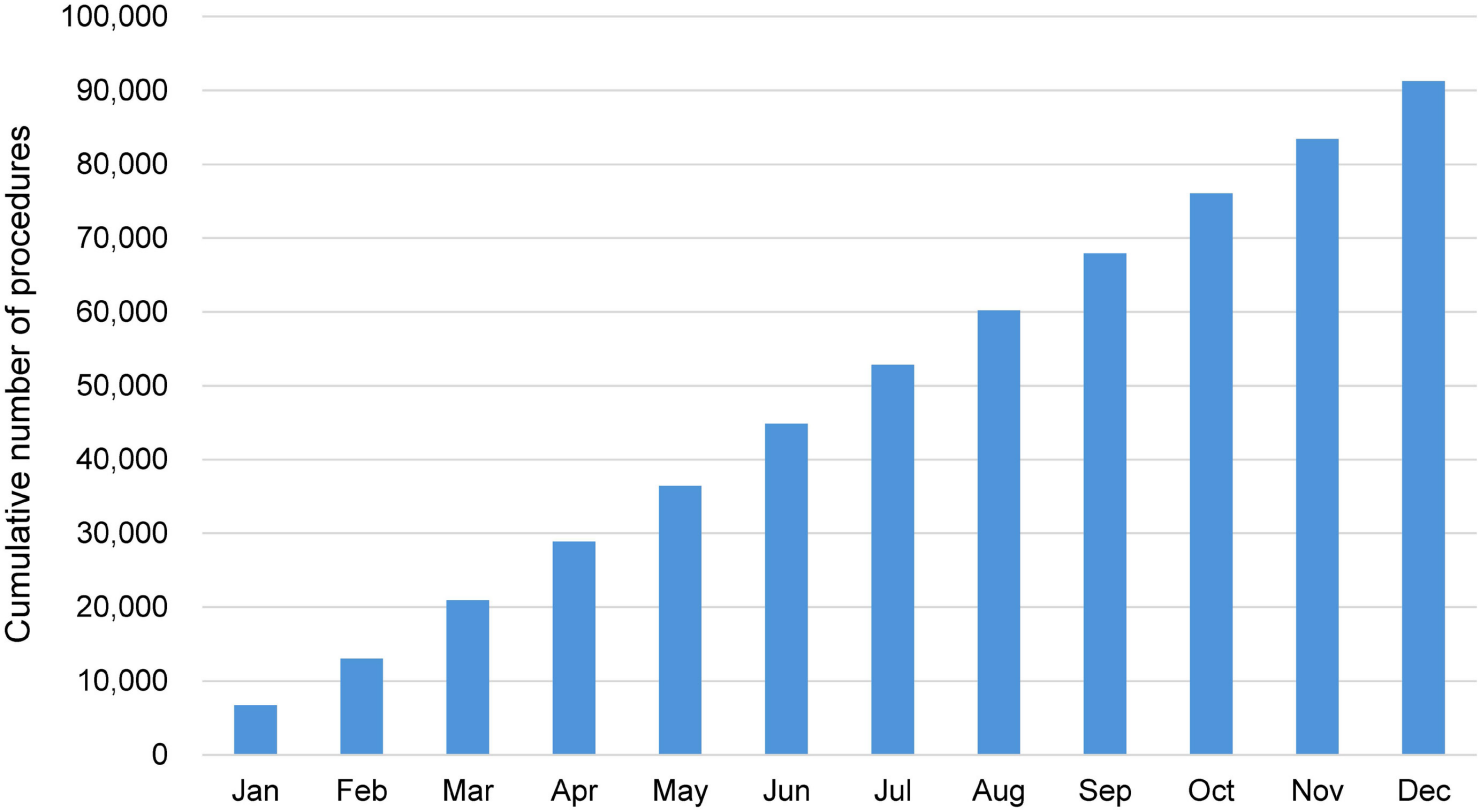
Cumulative number of registered hospitals (red line) and the patients (blue bars) during the year of 2022.

**FIGURE 2:**
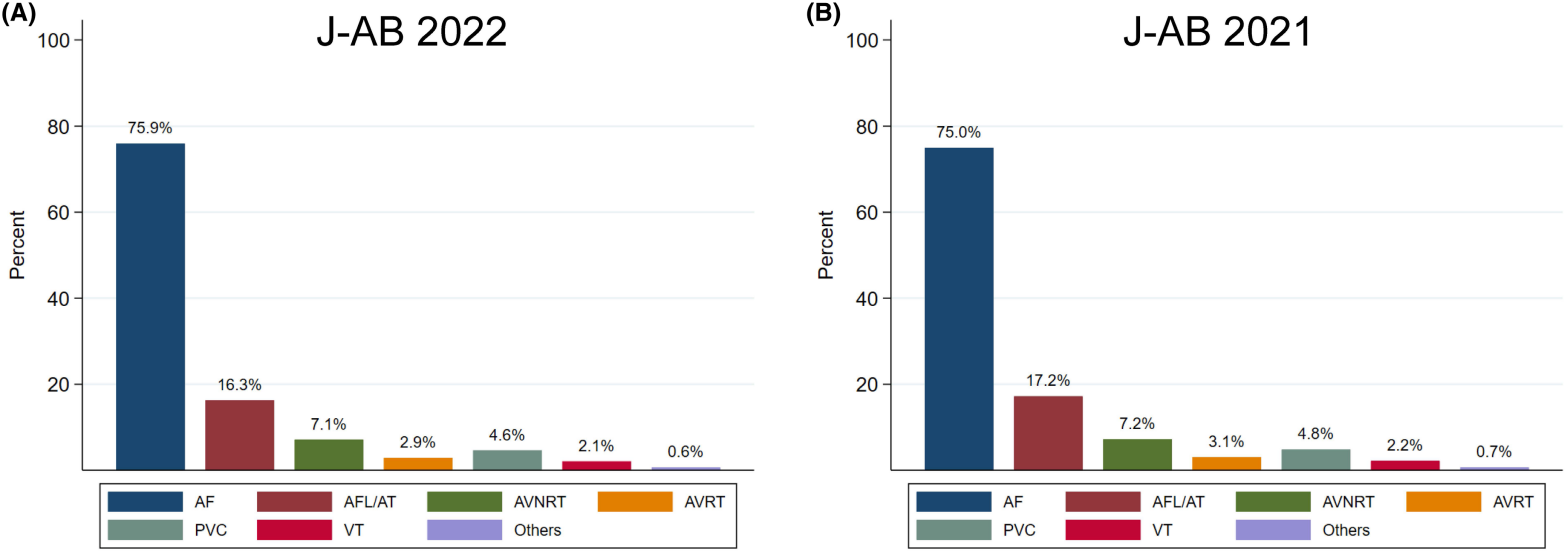
The number and rate of the target arrhythmias in the J‐AB 2022 (90,042 procedures; A) and 2021 (89,609 procedures; B). AF, atrial fibrillation; AFL, atrial flutter; AT, atrial tachycardia; AVNRT, atrioventricular nodal reentrant tachycardia; AVRT, atrioventricular reentrant tachycardia; IVC, inferior vena cava; PVC, premature ventricular contraction; VT, ventricular tachycardia; TV, tricuspid valve.

**TABLE 1:** Patient characteristics.

	All procedures	Atrial fibrillation (AF)			Atrial flutter (AFL)/Atrial tachycardia (AT)							Ventricular tachycardia (VT)		
		All AF	Paroxysmal AF (PAF)	Non‐PAF	All AFL/AT	IVC‐TV isthmus dependent AFL	Uncommon AFL macro AT	Focal AT	Atrioventricular nodal reentrant tachycardia	Atrioventricular reentrant tachycardia	Premature ventricular contraction	Idiopathic VT	VT because of ischemic cardiomyopathy	VT because of nonischemic cardiomyopathy
N	90,042	68,378	37,897	30,481	14,643	9292	3884	2797	6416	2584	4167	776	445	545
Age, mean ± SD	66.7 ± 13.0	68.3 ± 10.7	68.3 ± 11.1	68.4 ± 10.2	69.3 ± 12.5	69.5 ± 11.8	71.1 ± 11.9	66.7 ± 15.1	59.3 ± 16.7	48.9 ± 20.2	58.3 ± 16.5	56.9 ± 19.0	70.5 ± 9.5	63.5 ± 13.4
Gender, male	59,323 (65.9)	46,898 (68.6)	24,464 (64.6)	22,434 (73.6)	9871 (67.4)	7056 (75.9)	2238 (57.6)	1363 (48.7)	2708 (42.2)	1633 (63.2)	2347 (56.3)	509 (65.6)	407 (91.5)	433 (79.4)
Heart diseases														
IHD														
No	80,907 (89.9)	61,547 (90.0)	34,177 (90.2)	27,370 (89.8)	12,848 (87.7)	8086 (87.0)	3327 (85.7)	2583 (92.3)	6089 (94.9)	2495 (96.6)	3735 (89.6)	679 (87.5)	‐	498 (91.4)
Yes	7998 (8.9)	5975 (8.7)	3279 (8.7)	2696 (8.8)	1636 (11.2)	1115 (12.0)	504 (13.0)	189 (6.8)	255 (4.0)	57 (2.2)	395 (9.5)	88 (11.3)	‐	45 (8.3)
Unknown	1137 (1.3)	856 (1.3)	441 (1.2)	415 (1.4)	159 (1.1)	91 (1.0)	53 (1.4)	25 (0.9)	72 (1.1)	32 (1.2)	37 (0.9)	9 (1.2)	‐	2 (0.4)
Cardiomyopathy														
No	82,721 (91.9)	62,974 (92.1)	35,872 (94.7)	27,102 (88.9)	13,169 (89.9)	8384 (90.2)	3367 (86.7)	2573 (92.0)	6306 (98.3)	2526 (97.8)	3790 (91.0)	684 (88.1)	414 (93.0)	‐
Yes	6297 (7.0)	4619 (6.8)	1625 (4.3)	2994 (9.8)	1337 (9.1)	833 (9.0)	466 (12.0)	206 (7.4)	73 (1.1)	35 (1.4)	338 (8.1)	78 (10.1)	17 (3.8)	‐
Unknown	1024 (1.1)	785 (1.1)	400 (1.1)	385 (1.3)	137 (0.9)	75 (0.8)	51 (1.3)	18 (0.6)	37 (0.6)	23 (0.9)	39 (0.9)	14 (1.8)	14 (3.1)	‐
Valve disease														
No	83,609 (92.9)	63,606 (93.0)	35,873 (94.7)	27,733 (91.0)	12,869 (87.9)	8310 (89.4)	3074 (79.1)	2528 (90.4)	6251 (97.4)	2523 (97.6)	3995 (95.9)	733 (94.5)	382 (85.8)	480 (88.1)
Yes	5566 (6.2)	4082 (6.0)	1665 (4.4)	2417 (7.9)	1665 (11.4)	924 (9.9)	765 (19.7)	254 (9.1)	126 (2.0)	40 (1.5)	152 (3.6)	38 (4.9)	54 (12.1)	64 (11.7)
Unknown	867 (1.0)	690 (1.0)	359 (0.9)	331 (1.1)	109 (0.7)	58 (0.6)	45 (1.2)	15 (0.5)	39 (0.6)	21 (0.8)	20 (0.5)	5 (0.6)	9 (2.0)	1 (0.2)
CHD														
No	87,855 (97.6)	66,907 (97.8)	37,112 (97.9)	29,795 (97.7)	13,993 (95.6)	8900 (95.8)	3589 (92.4)	2695 (96.4)	6331 (98.7)	2526 (97.8)	4109 (98.6)	759 (97.8)	434 (97.5)	538 (98.7)
Yes	1224 (1.4)	695 (1.0)	376 (1.0)	319 (1.0)	542 (3.7)	334 (3.6)	249 (6.4)	89 (3.2)	42 (0.7)	39 (1.5)	32 (0.8)	12 (1.5)	2 (0.4)	5 (0.9)
Unknown	963 (1.1)	776 (1.1)	409 (1.1)	367 (1.2)	108 (0.7)	58 (0.6)	46 (1.2)	13 (0.5)	43 (0.7)	19 (0.7)	26 (0.6)	5 (0.6)	9 (2.0)	2 (0.4)

Abbreviations: CHD, congenital heart disease; IHD, ischemic heart disease; SD, standard deviation.

**TABLE 2:** Acute outcomes.

	2022	2021	2022–2021
	*n* (%)	*n* (%)	% change
Pulmonary vein isolation for atrial fibrillation	*n* = 67,967	*n* = 61,757	
Ablation system			
RF alone	49,416 (72.7%)	47,474 (73.1%)	−0.4
Balloon alone (Cryo, hot, laser)	13,399 (19.7%)	12,212 (18.8%)	+0.9
RF + Balloon combination	5083 (7.5%)	4979 (7.7%)	−0.2
Others	69 (0.1%)	417 (0.6%)	−0.5
Missing	‐	33 (0.05%)	
Patient with a first session	*n* = 55,170	53,113	
Success	54,960 (99.6%)	52,707 (99.2%)	+0.4
Unsuccess	210 (0.4%)	279 (0.5%)	−0.1
Unknown	0 (0.0%)	127 (0.2%)	−0.2
Patient with second session	*N* = 10,325	9623	
Success	7517 (72.8%)	7609 (79.1%)	−6.3
Unsuccess	31 (0.3%)	13 (0.1%)	+0.2
Already isolated	2777 (26.9%)	1950 (20.3%)	+6.6
Unknown	‐	51 (0.5%)	
Patient with ≥third session	*n* = 2445	2186	
Success	1137 (46.5%)	1226 (56.1%)	−9.6
Unsuccess	6 (0.2%)	3 (0.1%)	+0.1
Already isolated	1302 (53.3%)	948 (43.4%)	+9.9
Unknown	‐	9 (0.41%)	
IV‐TV isthmus‐dependent atrial flutter	*n* = 9292	*n* = 9605	
Success	9223 (99.3%)	9532 (99.2%)	+0.1
Unsuccess	69 (0.7%)	71 (0.7%)	0.0
Unknown	‐	2 (0.02%)	
Uncommon atrial flutter/atrial tachycardia	*n* = 3884	*n* = 3957	
Complete success	3322 (85.5%)	3392 (85.7%)	−0.2
Partial success	407 (10.5%)	366 (9.3%)	+1.2
Unsuccess	123 (3.2%)	135 (3.4%)	−0.2
Unknown	32 (0.8%)	64 (1.6%)	−0.8
Focal atrial tachycardia	*n* = 2797	*n* = 2894	
Complete success	2373 (84.8%)	2438 (84.2%)	+0.6
Partial success	298 (10.7%)	319 (11.0%)	−0.3
Unsuccess	89 (3.2%)	106 (3.7%)	−0.5
Unknown	37 (1.3%)	31 (1.1%)	+0.2
Atrioventricular nodal reentrant tachycardia by slow–fast	*n* = 5499	*n* = 5534	
Complete success	5377 (97.8%)	5418 (97.9%)	−0.1
Partial success	77 (1.4%)	80 (1.5%)	−0.1
Unsuccess	28 (0.5%)	21 (0.4%)	+0.1
Unknown	17 (0.3%)	15 (0.3%)	0.0
Atrioventricular nodal reentrant tachycardia by fast–slow	*n* = 607	*n* = 573	
Complete success	587 (96.7%)	542 (94.6%)	+2.1
Partial success	12 (2.0%)	17 (3.0%)	−1.0
Unsuccess	6 (1.0%)	10 (1.8%)	−0.8
Unknown	2 (0.3%)	4 (0.7%)	−0.4
Atrioventricular nodal reentrant tachycardia by slow–slow	*n* = 426	*n* = 356	
Complete success	402 (94.4%)	341 (95.8%)	−1.4
Partial success	19 (4.5%)	10 (2.8%)	+1.7
Unsuccess	3 (0.7%)	2 (0.6%)	+0.1
Unknown	2 (0.5%)	3 (0.8%)	−0.3
Atrioventricular reentrant tachycardia by kent	*n* = 2584	*n* = 2670	
Complete success	2461 (96.9%)	2586 (96.9%)	0.0
Unsuccess	51 (2.0%)	65 (2.4%)	−0.4
Unknown	28 (1.1%)	19 (0.7%)	+0.4
Premature ventricular contraction	*n* = 4167	*n* = 4314	
Complete success	3215 (77.2%)	3340 (77.4%)	−0.2
Partial success	718 (17.2%)	645 (15.0%)	+2.2
Unsuccess	198 (4.8%)	247 (5.7%)	−0.9
Unknown	36 (0.9%)	82 (1.9%)	−1.0
Idiopathic ventricular tachycardia	*n* = 776	*n* = 778	
Complete success	598 (77.1%)	616 (79.2%)	−2.1
Partial success	132 (17.0%)	126 (16.2%)	+0.8
Unsuccess	27 (3.5%)	25 (3.2%)	+0.3
Unknown	19 (2.4%)	11 (1.4%)	+1.0
Ventricular tachycardia because of ischemic cardiomyopathy	*n* = 445	*n* = 459	
Complete success	328 (73.7%)	320 (69.7%)	+4.0
Partial success	91 (20.4%)	97 (21.1%)	−0.7
Unsuccess	11 (2.5%)	24 (5.2%)	−2.7
Unknown	15 (3.4%)	18 (3.9%)	−0.5
Ventricular tachycardia because of nonischemic cardiomyopathy	*n* = 545	*n* = 570	
Complete success	314 (57.6%)	338 (59.3%)	−1.7
Partial success	183 (33.6%)	165 (29.0%)	+4.6
Unsuccess	26 (4.8%)	37 (6.5%)	−1.7
Unknown	22 (4.0%)	30 (5.3%)	−1.3

Abbreviations: IVC, inferior vena cava; RF, radiofrequency ablation; TV, tricuspid valve.

**TABLE 3:** Acute complications.

	2022		2021		2022–2021% change	
	All patient	AF	All patient	AF	All patient	AF
*N*	90,042	68,378	88,880	66,599		
Complications during hospitalization	2046 (2.27%)	1660 (2.43%)	2088 (2.35%)	1680 (2.52%)	−0.08%	−0.09%
Major bleeding (BARC ≥2)	772 (0.86%)	592 (0.87%)	813 (0.91%)	622 (0.93%)	−0.05%	−0.06%
Cardiac tamponade	496 (0.55%)	356 (0.52%)	473 (0.53%)	333 (0.50%)	+0.02%	+0.02%
Embolism	127 (0.14%)	106 (0.16%)	151 (0.17%)	130 (0.20%)	−0.03%	−0.04%
Phrenic nerve paralysis	293 (0.33%)	285 (0.42%)	261 (0.29%)	254 (0.38%)	+0.04%	+0.04%
Esophagus	119 (0.13%)	119 (0.17%)	129 (0.15%)	126 (0.19%)	−0.02%	−0.02%
Gastric hypomotility	114 (0.13%)	114 (0.17%)	116 (0.13%)	115 (0.17%)	0.0%	0.0%
Pericarditis	63 (0.07%)	56 (0.08%)	101 (0.11%)	79 (0.12%)	−0.04%	−0.04%
Sick sinus syndrome	125 (0.14%)	103 (0.15%)	179 (0.20%)	144 (0.22%)	−0.06%	−0.07%
Atrioventricular block	74 (0.08%)	20 (0.03%)	96 (0.11%)	31 (0.05%)	−0.03%	−0.02%
Death during hospitalization	118 (0.13%)	43 (0.06%)	93 (0.10%)	43 (0.06%)	+0.03%	0.0%
Cardiac death	69 (0.08%)	22 (0.03%)	43 (0.05%)	16 (0.02%)	+0.03%	+0.01%
Related to ablation therapy	2 (0.002%)	1 (0.001%)	1 (0.001%)	1 (0.002%)	0.0%	0.0%
Noncardiac death	49 (0.05%)	21 (0.03%)	49 (0.06%)	26 (0.04%)	−0.01%	−0.01%
Related to ablation therapy	2 (0.002%)	2 (0.003%)	1 (0.001%)	1 (0.002%)	0.0%	0.0%

## FUNDING INFORMATION

The funding for this study was provided by the Japanese Heart Rhythm Society.

## CONFLICT OF INTEREST STATEMENT

Kengo Kusano: Speaker honoraria from DAIICHI SANKYO COMPANY, Ltd., and Medtronic Japan, and research grants from Medtronic Japan, and JSR. Koichi Inoue: Speaker honoraria from DAIICHI SANKYO COMPANY, Ltd., Bristol Myers Squibb, Bayer Yakuhin, Nippon Boehringer Ingelheim, Johnson & Johnson KK, Medtronic Japan, and Boston Scientific Japan. Koji Miyamoto received research fundings irrelevant to this study from Abbott, Japan Lifeline, Boston, and lecture fees from Abbott, Nihon‐koden, Johnson & Johnson KK, Medtronic Japan, DAIICHI SANKYO COMPANY, Ltd., Brystol Myer Squibb, Pfizer, Bayer Yakuhin. Yasuo Okumura received research grants unrelated to this study from Johnson & Johnson KK and Biosense Webster, Inc., scholarship funds from Nippon Boehringer Ingelheim, remuneration from Daiichi‐Sankyo, AstraZeneca, Bayer Healthcare, Bristol‐Myers Squibb, and Johnson & Johnson KK, and belongs to the endowed departments of Boston Scientific Japan, Biotronik Japan, Abbott Medical Japan, Japan Lifeline, and Medtronic Japan. Kazuhiro Satomi received research fundings irrelevant to this study from Nippon Boehringer Ingelheim, Johnson & Johnson KK, Abbott Medical Japan, and lecture fees from Medtronic Japan, Japan Lifeline, DAIICHI SANKYO COMPANY, Ltd., Boston Scientific Japan, Bayer Yakuhin. Seiji Takatsuki belongs to Advanced Cardiac Arrhythmia Therapeutics Endowed Research Course, which is supported by Medtronic Japan, Japan Lifeline, Boston Scientific Japan, Abbot Japan and Biotronik Japan. He received research fundings irrelevant to this study from Nippon Boehringer Ingelheim, Japan Lifeline, Boston Scientific Japan, and lecture fees from Medtronic Japan, Japan Lifeline, DAIICHI SANKYO COMPANY, Ltd., Boston Scientific Japan, Bayer Yakuhin, Biotronik Japan, Abbot Japan, and Nihon‐koden. Teiichi Yamane: Speaker honoraria from Medtronic Japan, Johnson and Johnson KK, and BEG company, and research grants from Japan Lifeline. Wataru Shimizu: Speaker honoraria from DAIICHI SANKYO COMPANY, Ltd., Nippon Boehringer Ingelheim, Pfizer, Johnson & Johnson KK, Boston Scientific Japan, Japan Lifeline, Medtronic Japan, and Abbott. None: K.K., Y.I., K.N, and Y.I.

## ETHICS STATEMENT

This study was approved by the central ethics review board of the Japanese Heart Rhythm Society (No. 2021001, approved on Dec 16, 2021).
